# Modified Titanium Implant as a Gateway to the Human Body: The Implant Mediated Drug Delivery System

**DOI:** 10.1155/2014/801358

**Published:** 2014-07-22

**Authors:** Young-Seok Park, Joo-Youn Cho, Shin-Jae Lee, Chee Il Hwang

**Affiliations:** ^1^Department of Oral Anatomy, Seoul National University School of Dentistry and Dental Research Institute, Seoul 110-749, Republic of Korea; ^2^Department of Oral and Maxillofacial Diagnostic Sciences, University of Florida College of Dentistry, Gainesville, FL 32610, USA; ^3^Department of Pharmacology and Clinical Pharmacology, Seoul National University College of Medicine, Seoul 110-744, Republic of Korea; ^4^Department of Orthodontics, Seoul National University School of Dentistry and Dental Research Institute, Seoul 110-768, Republic of Korea; ^5^Hwang Chee Il Dental Office, Incheon 402-201, Republic of Korea

## Abstract

The aim of this study was to investigate the efficacy of a proposed new implant mediated drug delivery system (IMDDS) in rabbits. The drug delivery system is applied through a modified titanium implant that is configured to be implanted into bone. The implant is hollow and has multiple microholes that can continuously deliver therapeutic agents into the systematic body. To examine the efficacy and feasibility of the IMDDS, we investigated the pharmacokinetic behavior of dexamethasone in plasma after a single dose was delivered via the modified implant placed in the rabbit tibia. After measuring the plasma concentration, the areas under the curve showed that the IMDDS provided a sustained release for a relatively long period. The result suggests that the IMDDS can deliver a sustained release of certain drug components with a high bioavailability. Accordingly, the IMDDS may provide the basis for a novel approach to treating patients with chronic diseases.

## 1. Introduction 

In general, drug delivery systems are designed to effectively deliver the required drug amounts while maximizing the efficacy and effectiveness of drugs and minimizing their side effects. The new drug delivery systems and technologies that are currently being developed promise to make the administration of medicines more efficient and less painful [1]. The efficacy and effectiveness of drug delivery systems are of particular importance to chronic illness sufferers, who often require drugs to be continuously administered for long periods.

The most common type of drug administration is oral administration. However, patients often fail to take orally administered drugs regularly when they are prescribed for long periods. For some pharmacological agents, more direct therapeutic approaches are required to bypass the gastrointestinal barrier and deliver the drugs directly into the blood stream. A common alternative is to administer periodic injections through devices such as the insulin pump used by diabetics who require daily injections of insulin. However, the insulin pump employs an injection needle and the frequent injections are poorly tolerated by patients. There patients often experience pain, fear, and unnecessary limitations to daily life, which can be considerable inconvenience. These drawbacks have led to the development of alternative delivery systems to needle injections [1, 2].

An effective alternative drug delivery system needs to address the natural question of how to avoid painful yet frequent needle injections and maintain the drug efficacy while requiring minimal long-term compliance from patients. To reduce the aforementioned inconveniences while maximizing drug efficacy, new drug delivery systems need to be developed that can control the release of drugs and reduce the number of drug administrations. In the field of ophthalmology, the implantation of sustained drug release devices in the eye has been proposed as an alternative option [1, 3, 4]. However, the frequent surgical placement and removal of drug-containing implants is not practical for the routine management of chronic diseases. The new drug delivery system proposed in this study is a specially designed nonabsorbable implant that is capable of acting as a gate into the body. This permanent gateway can provide patients with sustainable drug release when required over long periods without requiring multiple needle injections or frequent drug uptake.

The use of titanium implants has become increasingly widespread and is gaining popularity, especially in contemporary dentistry [5–7]. Recent studies have demonstrated that titanium implants have a reliable success rate due to their well-documented biocompatibility [8–11]. Various types of such implants are widely used today to substitute for missing teeth and to act as supporting retentive structures or anchorage devices [7, 12–16].

The aim of this study was to examine the efficacy and feasibility of the proposed new implant mediated drug delivery system (IMDDS), which does not require frequent oral administration or painful needle injections. A pharmacokinetic study using dexamethasone was conducted on rabbits to investigate how the IMDDS works in an animal model.

## 2. Materials and Methods

### 2.1. Animal Subject

Fourteen New Zealand white male rabbits weighing 2.5–3 kg were prepared as the experimental animal. The rabbits were kept in separate cages and fed a standard rabbit diet. The selection, care, surgical protocol, and preparation of the animals were all conducted according to the guidelines of the Institutional Animal Care and Use Committee of the Seoul National University School of Dentistry. The internal review board approved the protocol for the rabbit experiments in this study (IRB no. SNU-140103-3).

### 2.2. The Modified Implant Design

**Figure 1:**
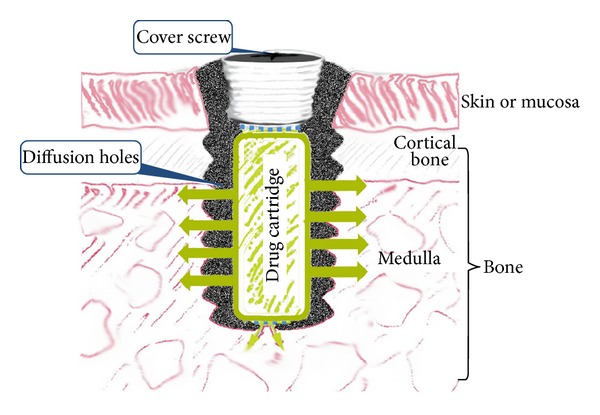
The implant used in the IMDDS comprised several components: a hollow titanium implant configured to be implanted in bone, an accommodating part formed therein to allow a drug cartridge to be seated, multiple diffusion holes formed in the circumferential wall of the implant, and a cover screw on top of the implant to close the gate of the IMDDS.

Threaded implants were custom made by machining a block of pure titanium (grade 4). The implants used in the IMDDS comprised several components: an implant configured to be implanted in the bone, an accommodating part formed therein to allow a drug cartridge or a drug cassette to be seated within the implant, multiple diffusion holes formed in the circumferential wall of the implant from which the content of the drug cartridge can penetrate and disperse into the body system, and a cover unit coupled to the implant that closes the gate of the IMDDS (Figure 1).

### 2.3. Surgical Procedure

**Figure 2:**
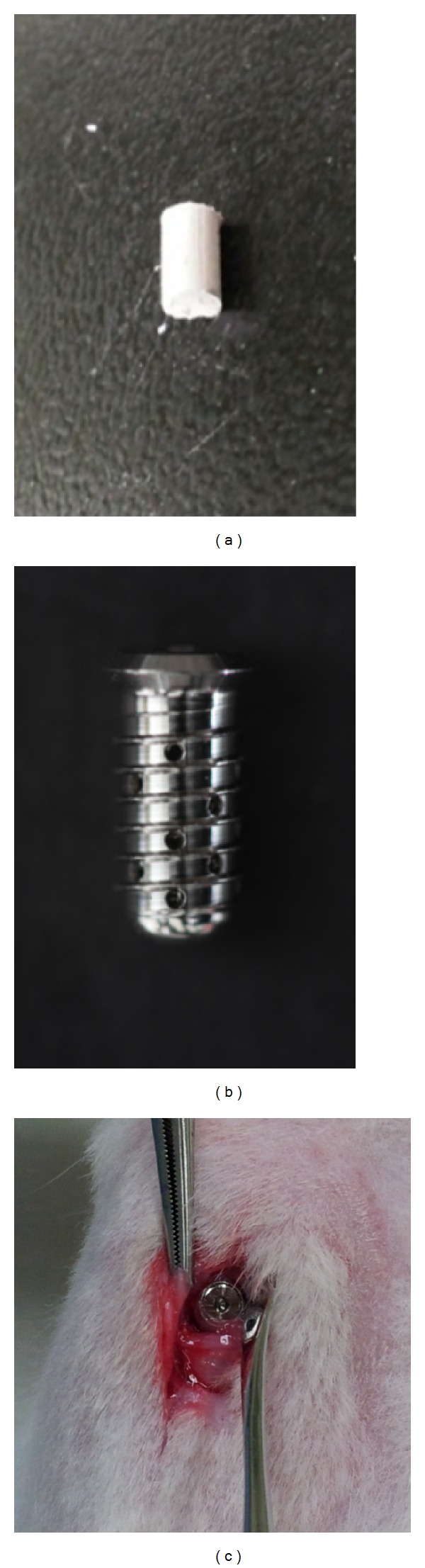
The dexamethasone cartridge (a), the cartridge inserted into the implant (b), and the implant placed in the rabbit tibia (c).

During the implant placement, general anesthesia was induced by the intramuscular injection of 10 mg/kg of Zoletil (Virbac) and 0.15 mL/kg of Rompun (Bayer Korea, Seoul, Korea). Prior to surgery, the skin in the mesial proximal tibia was shaved and then washed with an iodine solution. A preoperative antibiotic (0.15 g kanamycin intramuscularly) was also administered prophylactically. One milliliter of a 2% lidocaine solution with 1 : 100,000 epinephrine was injected into the region of the planned surgery. A periosteal incision was made to expose the tibia. After dissecting the muscles and periosteum, the flat surface on the lateral aspect of the proximal tibia was selected for implant placement. A low-speed rotary engine was used to drill the hole for the implant under profuse irrigation with sterile saline. Each rabbit received one implant in the tibia. The entire surgery procedure was performed under sterile conditions to prevent infection. After surgery, each rabbit received an intramuscular injection of antibiotics (Figure 2). A more detailed description of the procedure is available in the previous publication by Lee et al. (2009) [17].

### 2.4. Pharmacokinetic Investigation

Pharmacokinetic studies are crucial for understanding drug delivery systems. Such studies involve measuring the concentration of a drug in plasma or blood at several time points after drug administration [18].

In this study, a pharmacokinetic investigation was performed using an administration of dexamethasone, which has well-established pharmacokinetic properties [19, 20]. Dexamethasone is an efficient anti-inflammatory drug used in the treatment of several chronic diseases.

The dexamethasone powder (D1756-1G, Sigma-Aldrich, St Louis, MO) was prepared in 23 mg cartridges. A single cartridge of dexamethasone was inserted within the implant and sealed with a cover screw (Figure 2).

### 2.5. Experimental Design and the Measurement of the Dexamethasone Concentration

Three-milliliter blood samples were taken from the marginal vein of the ear at predetermined time intervals from immediately after the dexamethasone administration up to the longest follow-up time of 15 weeks (3 months and 2 weeks). The blood samples were collected in heparin tubes and divided into two 1.5 mL tubes for centrifugation. The plasma was taken for analysis after separation via centrifugation (3,000 rpm, 10 minutes in 4°C). The plasma concentration of the dexamethasone was determined using liquid chromatography tandem mass spectroscopy (LC-MS/MS System, AB SCIEX, Framingham, MA) at each time point.

Considering the ethics of animal experimentations and the restricted rabbit blood volume, a batch experimental design was used for the sampling schedule instead of the classic complete data design. Unlike the complete data design, which samples each subject at all predefined time points, the batch design takes samples more than once from each subject, but not at all time points [18].

To distinguish the sustained release of the dexamethasone from the implant from the carry-over effect of dexamethasone metabolism, the implants were removed from two of the rabbits. The blood sampling was then repeated 2 weeks after the removal of the implants.

### 2.6. Statistical Data Analysis

The R programming language [21] and the R package PK [22] were used to perform the data analysis. The area under the concentration versus time curve (AUC) for the dexamethasone concentration was calculated.

## 3. Results

There were no abnormalities, mobility, or inflammation on the implant sites up to 8 months after the placement of the implants.

The cumulative release profiles of dexamethasone were obtained for 12 of 14 rabbits, with the 2 other rabbits being used for pilot experiments. Considerable variation was observed in the dexamethasone concentrations in each experimental rabbit. However, two common features were observable. First, the release profile demonstrated no lag time immediately after the dexamethasone administration. Second, a sustained release pattern was observed up to 7 weeks after administration. After 10 weeks, the dexamethasone was not detectable.

**Figure 3:**
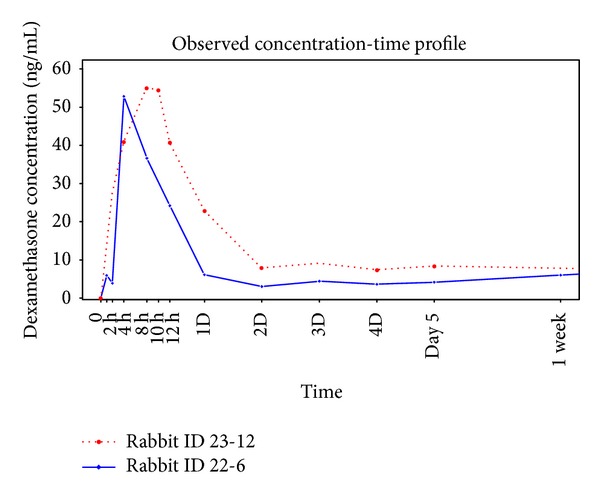
Dexamethasone release profiles of the 2 representative rabbits (rabbits ID 22-6 and ID 23-12). There was no lag period immediately after dexamethasone administration. During the first day after the drug cartridge insertion, a considerable amount of the drug was released and the maximum concentration was detected at between 4 hours and 12 hours after administration. From the second day, a sustained release was maintained and the concentration provided relatively constant levels of dexamethasone for more than 7 weeks.

To concisely describe the results, the release profiles of two selected rabbits were depicted in Figure 3. During the first day after the drug cartridge insertion, a considerable amount of the drug was released and the maximum concentration was detected at between 4 hours and 12 hours after administration (Figure 3). From the second day, a sustained release of dexamethasone was maintained and relatively constant levels were provided for more than 7 weeks.

**Figure 4:**
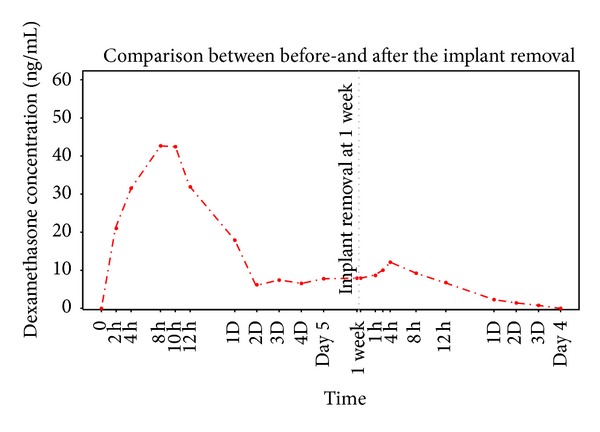
Comparison of the dexamethasone release profile before and after the implant removal. After the removal, as the drug depleted, the dexamethasone release rate declined sharply. Four days after the implant removal, dexamethasone was not detectable.

The biological half-life of dexamethasone is relatively long, at 36–54 hours in rabbits [19, 20]. The prolonged detection may have been the result of the IMDDS's sustained release pattern or a carry-over effect of the drug in rabbits. To distinguish the cause of the sustained release, we removed the implants from 2 rabbits at 1 week after drug administration. Again, the dexamethasone concentration was evaluated for up to 1 week (Figure 4). After the removal, as the drug depleted, the dexamethasone release rate declined sharply. No later than 4 days after the implant removal, dexamethasone was not detectable. This finding suggests that the sustained detection was unlikely to have been influenced by the long half-life of dexamethasone in the rabbit body.

The AUC is a reliable index for estimating the bioavailability of drugs [3]. Calculation of the areas under the curve revealed that the total AUC was 12.0 mg/mL. The AUCs for each experimental unit demonstrated that the longer the follow-up time, the greater the AUC (Table 1). To restate, the drug release pattern of the IMDDS showed a sustained release for a relatively long period. Based on the AUC, the bioavailability of dexamethasone in the rabbits treated with the IMDDS appeared evident.

## 4. Discussion 

All of the inserted implants remained intact without any complication in their original position during and after the experiment. This result is not surprising, because the stability and biocompatibility of titanium implants have been reported to be almost perfect [5, 11, 13]. In fact, the high survival rate of titanium implants is not greatly influenced by clinical factors. For example, characteristics such as whether the site of implantation was located in the mandible or in the maxilla [6, 7], anywhere in the oral cavity, or in extremities such as the rabbit tibia; whether the implant length was short or long; or whether the diameter was large or small did not affect the high survival rate [9, 10, 13]. Accordingly, it is unlikely to be biocompatibility issues with the titanium implants used in the IMDDS. Because the implants for the IMDDS come into direct contact with the body, the implants must be made from materials that are biocompatible and pharmacologically inert.

To the best of our knowledge, this study is the first to report a drug delivery system using a titanium implant. The proposed idea for the study was simple, to examine whether a modified dental implant is capable of providing a gate for delivering drugs continuously for a long period. The dexamethasone delivery using the IMDDS demonstrated promising results in the rabbit experiment. The findings also suggest that drug delivery by the IMDDS is relatively steady over a relatively long time. From the sustained drug release profile, it can be clearly inferred that the IMDDS allowed a constant release of the drug without any apparent lag phase or conspicuous initial burst. Sustained release systems are very useful for the long-term administration of drugs because a single administration can achieve the same effect as multiple doses.

Several chronic diseases are often treated with repeated needle injections to maintain drug concentrations. Furthermore, if the drugs have a short half-life, multiple repeated injections are required. However, repeated injections over an extended period to ensure therapeutic levels often lead to reduced patient compliance or an increased likelihood of complications [3, 23]. The search for a slow or sustained release drug delivery system situated in the ocular area led to the development of a biodegradable intraocular implant [4], which has been introduced in the field of ophthalmology. The intraocular implant, which was designed to release dexamethasone for an extended period at a steady rate, was developed to treat inflammation after cataract surgery to decrease the risk of systemic toxicity and ocular side effects [3, 4, 24].

If the direct administration of drugs to a specific localized lesion is required [25], placing the implant as close as possible to the lesion could be more efficient. This could provide a more direct way of achieving the required therapeutic concentration through the local anatomy. In this regard, the IMDDS may provide a more satisfactory means of access than the systemic administration of drugs through the blood vessels or oral uptake. Moreover, placing the implant at the site closest to the target lesion may enable more direct administration, which may also prevent the adverse effects associated with systemic administration. The IMDDS would be beneficial in this case because it is capable of providing a fast, more direct, and sustained release of the therapeutic agent to local bone areas. The intraosseous application of drugs can also bypass the systemic circulation, allowing for the accumulation of higher intrabony drug concentrations than can be achieved by systemic or surface administration.

The anatomic and physiological differences between rabbits and humans should be considered before applying the findings of this study to humans. The past findings for rabbits should also be differentiated from what may happen in humans. For example, rabbits have a smaller body mass; it has been shown that a dose of 25 mg dexamethasone in rabbits is equivalent to 500 mg in a 70 kg man [2]. Accordingly, the drug concentrations measured in rabbits tend to be significantly higher than those recorded in humans. For potential human applications, a thorough understanding of the pharmacokinetics of the IMDDS is necessary.

Implementing the IMDDS for clinical use may also be challenging. As with any new technique, clinicians would require training and education on the use of the new drug delivery system. Because the IMDDS is not 100% safe, skilled clinicians would be needed to administer the system. We do not anticipate that the IMDDS would be used by home use patients who self-manipulate, as the drug delivery system is designed for professional use only. To maintain or reassemble the drug cartridge or cassette, patients would need to visit a trained clinician such as a dentist.

Although we were not able to directly determine the efficiency of the IMDDS in this study, we found that the implant had satisfactory stability without inflammation and obtained promising pharmacokinetic characteristics using an animal experiment. However, further research is necessary to achieve a more controlled drug release via the IMDDS. A lag period or initial burst immediately after drug administration is common in oral and injection administrations. Although the IMDDS did not show a lag time, a delayed type of initial burst was observed. This could be controlled by the physicochemical properties of the cartridge to guarantee sustained release. Whether the drug preparation is a gel type or a powder type may influence the duration of the release and the peak concentration. It may also be possible to develop an electronic smart module that can control the release pattern of the IMDDS and monitor the drug release. The drug concentration may vary with factors such as the type and material of the drug cartridge, the implantation site, and the viscosity and solubility of the drug components. Through several alterations of these factors, the IMDDS would be a versatile drug delivery system that can provide the controlled sustained release of drugs.

The IMDDS is currently in the early stages of development. The major advantages of the IMDDS are the elimination of broken needles and a more constant delivery of drugs with minimal patient compliance. The implants for the IMDDS are customizable to each anatomical location of the body and can be modified to optimize the efficiency and efficacy of the system. The implantation site is a crucial element in ensuring that a proper dosage is released in recipients. There may be as yet unknown variables that prevent proper dosing with the IMDDS. Proper administration is highly dependent on numerous possible factors. Therefore, it may be pragmatic to develop a more convenient drug cartridge, such as a disposable type of drug cassette. This would have the added advantage of enabling a variety of different doses or different drugs to be loaded in the implant for use in the treatment of various diseases.

We envision that the IMDDS will be capable of providing a relatively safe method of administrating prolonged therapeutic levels of drugs. We hope that the IMDDS will provide an alternative to the existing needle-based drug delivery systems for chronic sufferers.

## 5. Conclusions

We conducted the first investigation of a drug delivery system using a modified titanium implant based on a study of the pharmacokinetics of dexamethasone in rabbits. To examine the efficacy and feasibility of the proposed IMDDS, we investigated the pharmacokinetic behavior of dexamethasone in plasma after delivering a single dose via the modified implant placed in the rabbit tibia. Our results indicate that drug delivery using the modified dental implant has a number of promising features. In particular, the IMDDS allows sustained drug delivery with a prolonged duration of drug action and a high bioavailability, therefore providing the basis for a novel approach to treating patients with chronic diseases. The chief advantage of the system is that no repeated needle injections or timely oral uptakes are necessary to maintain the critical drug concentrations. Although further experiments are necessary, the IMDDS shows great promise for the treatment of chronic diseases that require repeated drug administration or when timely periodic drug uptake is of the utmost importance.

## Figures and Tables

**Table 1:** Area under the curve (AUC) of the plasma concentration of dexamethasone.

Experimental units	Number of rabbits per unit batch, *n*	Maximum follow-up time	AUC (mg/mL)
Batch number 1	4	3 months and 2 weeks	16.69 (7.33)
Batch number 2	2	7 weeks	2.83 (0.39)
Batch number 3	2	7 days	1.48 (0.74)
Batch number 4	4	1 day	1.20 (0.23)
Pooled data	*N* = 12		12.00 (1.58)

The values in parentheses are the standard errors.
